# Ball-Milled Recycled Lead-Graphite Pencils as Highly Stretchable and Low-Cost Thermal-Interface Materials

**DOI:** 10.3390/polym10070799

**Published:** 2018-07-20

**Authors:** Chun-An Liao, Yee-Kwan Kwan, Tien-Chan Chang, Yiin-Kuen Fuh

**Affiliations:** 1 Department of Mechanical Engineering, National Central University, No. 300, Jhongda Rd., Jhongli City, Taoyuan County 32001, Taiwan; kenksw@hotmail.com (C.-A.L.); m456123a@yahoo.com.tw (Y.-K.K.); 2 Institute of Nuclear Energy Research, Atomic Energy Council, Executive Yuan, No. 1000, Wenhua Rd., Jiaan Village, Longtan Township, Taoyuan County 32546, Taiwan; market1210@gmail.com

**Keywords:** thermal-interface materials (TIMs), ball milling, recycled lead-graphite pencils, thermal conductivity

## Abstract

A simple and sustainable production of nanoplatelet graphite at low cost is presented using carbon-based materials, including the recycled lead-graphite pencils. In this work, exfoliated graphite nanoplatelets (EGNs), ball-milled exfoliated graphite nanoplatelets (BMEGNs) and recycled lead-graphite pencils (recycled 2B), as well as thermally cured polydimethylsiloxane (PDMS), are used to fabricate highly stretchable thermal-interface materials (TIMs) with good thermally conductive and mechanically robust properties. Several characterization techniques including scanning electron microscopy (SEM) and thermogravimetric analysis (TGA) showed that recycled nanoplatelet graphite with lateral size of tens of micrometers can be reliably produced. Experimentally, the thermal conductivity was measured for EGNs, BMEGNs and recycled 2B fillers with/without the effect of ball milling. The in-plane thermal conductivities of 12.97 W/mK (EGN), 13.53 W/mK (recycled 2B) and 14.56 W/mK (BMEGN) and through-plane thermal conductivities of 0.76 W/mK (EGN), 0.84 W/mK (recycled 2B) and 0.95 W/mK (BMEGN) were experimentally measured. Anisotropies were calculated as 15.31, 15.98 and 16.95 for EGN, recycled 2B and BMEGN, respectively. In addition, the mechanical robustness of the developed TIMs is such that they are capable of repeatedly bending at 180 degrees with outstanding flexibility, including the low-cost renewable material of recycled lead-graphite pencils. For heat dissipating application in high-power electronics, the TIMs of recycled 2B are capable of effectively reducing temperatures to approximately 6.2 °C as favorably compared with thermal grease alone.

## 1. Introduction

Significant thermal management difficulties occur in many high-density microelectronic devices ubiquitous in many communication and energy storage applications [1,2], high-power devices of insulated gate bipolar transistors (IGBTs) [1], gallium nitride (GaN) light-emitting diodes and field-effect transistors (FETs) [3,4,5,6]. Among the strategies dedicated to thermal management, thermal-interface materials (TIMs) [7] are widely applied as effective heat dissipation passages such as those massively produced in solar cells [8,9] and electronics signal wires or electroplated electrodes [3,10]. Considering the trade-off between production cost and material properties (mechanical, chemical and thermal attributes), the primarily used matrix consists of epoxy-based resins [4,5,6] with graphite nanoplatelet materials [11] to reduce the cost of embedded nanoscale fillers [12,13]. In addition, enhancing the heat dissipating performance of TIMs by using thermally conductive nanomaterial fillers is one of the thrust areas, for example, carbon-based materials (carbon nanotubes, carbon fibers, carbon nanosheets [14], exfoliated graphite nanoplatelets (EGNs), graphene [15,16,17,18], NiO/graphene [19] and FeO(OH)/activated carbon [20]). Furthermore, phase-change materials using exfoliated graphite with high shape stabilization [17] and other novel nanostructured composites such as MnO_2_/graphene [21] and Co_3_O_4_/graphene [22] have been applied in microelectronic electrode materials.

Concerning the TIMs’ manufacture, the additive-type patterning of screen-printing technology [23,24,25] is widely used for EGN-based composites. Thermally cured and screen-printed EGN/SNP-embedded polydimethylsiloxane (PDMS) TIMs and ball milling was investigated in [26,27]. Moreover, ball milling of nanofillers and mechanically mixing into a polymer matrix [28], which is an environmentally and economically sustainable method [29,30,31], was investigated at low cost in the mass-production process [32,33,34]. A novel ultrasonicated ozonolysis (USO) processing method has been applied to make highly stable aqueous dispersions of exfoliated graphite (EG), which can be processed for ink-jet printing [35].

In this research, a simple and convenient means to physically disintegrate recycled lead-graphite pencils (2B graphite, Pentel Co., Ltd., Taoyuan, Taiwan) was experimentally examined by using a ball-mill machine (Mixer Mills, Retsch MM400, Nürnberg, Germany). A previous study [36] indicated that the increase of the specific surface area can effectively promote the interaction among particles, and thus, improve the effective thermal conductivity. Screen-printing technology is used to make the composite film ~0.2 mm thick. Experimental measurements were systematically investigated to characterize the recycled nanoplatelet graphite-embedded polydimethylsiloxane (PDMS) TIMs (recycled 2B). Finally, the thermal conductivity and the application to IGBT were investigated. Experimental results indicate that TIMs with low loading of functionalized EGN and recycled nanoplatelet graphite fillers, which can be compliantly oriented during the composite application to the surfaces, have great potential in thermal management of advanced electronics.

## 2. Materials and Methods

### 2.1. Materials and Preparation of Recycled 2B TIMs

**Figure 1:**
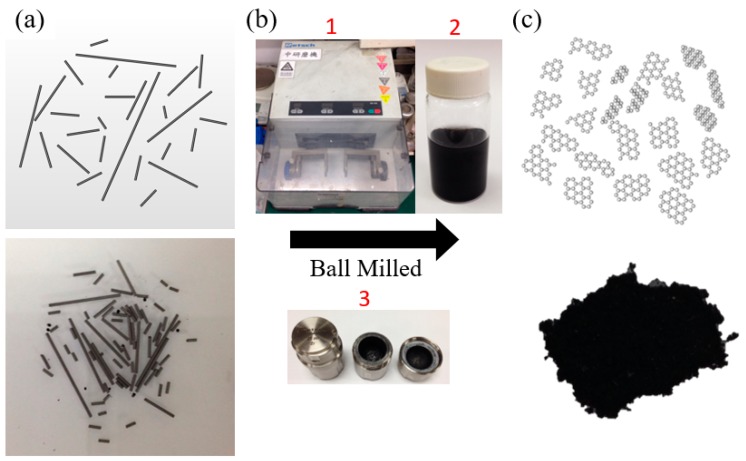
A schematic representation of (**a**) recycled 2B; (**b**) optical photo showing the mechanical setup to produce nanoplatelet graphite from recycled pencil lead by ball milling where (1) is the ball-mill machine; (2) suspension of the recycled nanoplatelet graphite in the ethanol medium; (3) 25 mL ball-mill jar; and (**c**) recycled nanoplatelet graphite.

A schematic representation and the optical image of recycled nanoplatelet graphite by ball-milling of recycled 2B is shown in Figure 1a,b; Figure 1c shows the optical photo and schematic of disintegration of recycled nanoplatelet graphite via the solid-state ball-milling approach [26] such that continuous mechanochemical fragmentation and associated crystallite sizes can be exfoliated to the nanometer range.

Experimentally, recycled 2B was selected in this study for the filler material, together with commercially available nanoplatelet graphite. Initially, the ball milling of recycled 2B to produce recycled nanoplatelet graphite was carried out in a ball-mill machine (Retsch MM400, Haan, Germany). 2.0 g of recycled 2B was typically charged into a stainless steel capsule (25 mL) with zirconia beads of 3 mm in diameter. The container was vibrated at a frequency of 20 Hz for durations of approximately 4 h, then carefully collected and washed with the solution of water and ethanol. The final products were dried in vacuum oven at 60 °C under a reduced pressure (~93 kPa) for 4 h to yield 1.08 g of recycled 2B. The ball-milling process is performed at dry ambient environment.

**Figure 2:**
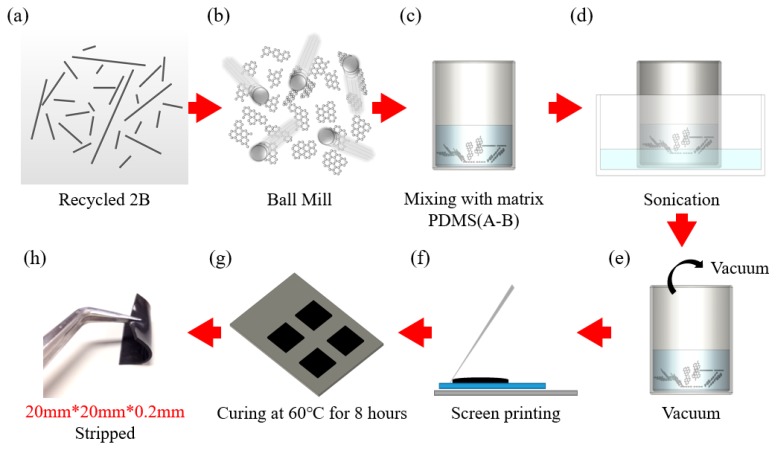
(**a**) Digital optical photo of recycled 2B as prepared; (**b**–**g**) schematic of sample preparation route for the recycled 2B; (**h**) shows the excellent flexibility of fabricated TIMs.

The schematic of the screen-printing technique to functionally facilitate the spreading and bonding process of TIMs onto an aluminum heat sink in high-power applications is presented in Figure 2. Specifically, Figure 2a shows the images of recycled 2B powder which were obtained with a common digital camera. Figure 2b shows the fillers (EGN, recycled 2B and BMEGN) with the ball-milled powders. In order to fabricate the composite fabrication, the PDMS prepolymer (Sylgard 184A) and recycled 2B were first spatula-dispersed and magnetically agitated for 1 h at 100 rpm. Next, PDMS curing agent (Sylgard 184B) was thoroughly (respecting a 10:1 ratio between the prepolymer and curing agent) incorporated into the mixture (Figure 2c), using a conventional sonication at power of 100 W for 3 h at room temperature (Figure 2d) and 30 min degassing (Figure 2e). To screen print composite structures on a glass substrate, the well-mixed pastes (EGN, recycled 2B and BMEGN) were initially forced into the glass substrate using a 45° angle tilt (Figure 2f). After the screen-printing process, the samples were oven-heated at 60 °C for 8 h and mechanically stripped off (Figure 2g).

### 2.2. Analysis Methods 

The morphologies of EGN, recycled 2B and BMEGN were analyzed by scanning electron microscopy (FE–SEM, S-4800, HITACHI, Tokyo, Japan) at room temperature and atmospheric conditions. Thermogravimetric analysis (TGA) was measured in the temperature range from 50 to 1000 °C with a 200 °C/min ramp rate and a 4 °C/min resolution using TG–DTA (10 °C/min, Perkin Elmer TGA-7, Waltham, MA, USA). The through-plane thermal conductivity was measured by a plane heat source (hot plate) method as described by ASTM D5470-06 [37] with the equipment in the experimental setup consisting of an electrical heater, a heat sink and two thermocouples to measure the temperature gradient [11]. The in-plane thermal conductivity was measured utilizing a comparative technique [38]. The in-plane thermal conductivities of the EGN, recycled 2B and BMEGN composites were measured in the temperature range 30–40 °C by utilizing a comparative technique, similar to the measurement setup of the previous study [38].

## 3. Results and Discussions

### 3.1. Mechanical Properties

**Figure 3:**
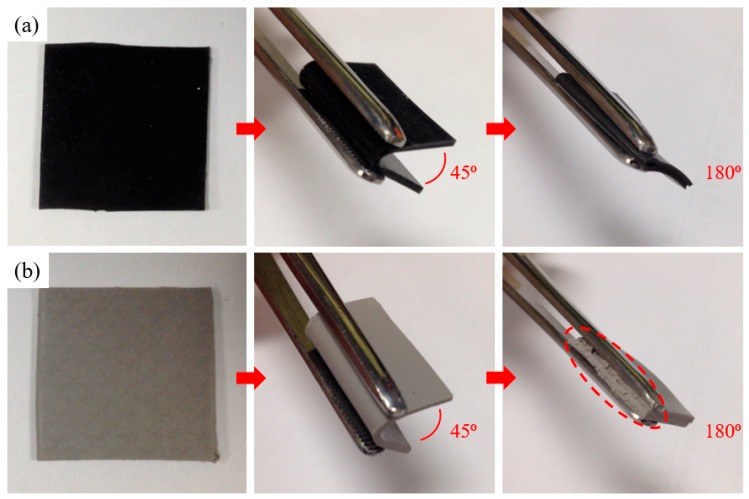
Optical photos of (**a**) recycled 2B composite, compared with (**b**) a commercial TIM. The recycled 2B composite is experimentally shown to be mechanically flexible and structurally robust after bending 180 degrees by tweezer. (Samples’ dimensions: 20 mm × 20 mm × 1 mm)

The recycled 2B composite (10 vol % recycled 2B loading) is black (Figure 3a) and mechanically demonstrates the high degree of flexibility, compared favorably with the commercially available thermal-interface material counterpart (Figure 3b, TG-6050). The sample was commercially available from T-Global Technology Co., Ltd., Yilan, Taiwan. After bending 180 degrees, the commercial TIMs showed severe cracks while the proposed counterpart of recycled 2B composite was mechanically flexible and structurally robust after repeated cycling tests (~100 cycles). In summary, the preparation and mechanical characteristics of proposed recycled 2B composite films were comparatively superior to those of commercially available TIMs, and furthermore, the thermal conductivities will be compared in the next section.

### 3.2. Scanning Electron Microscopy (SEM)

**Figure 4:**
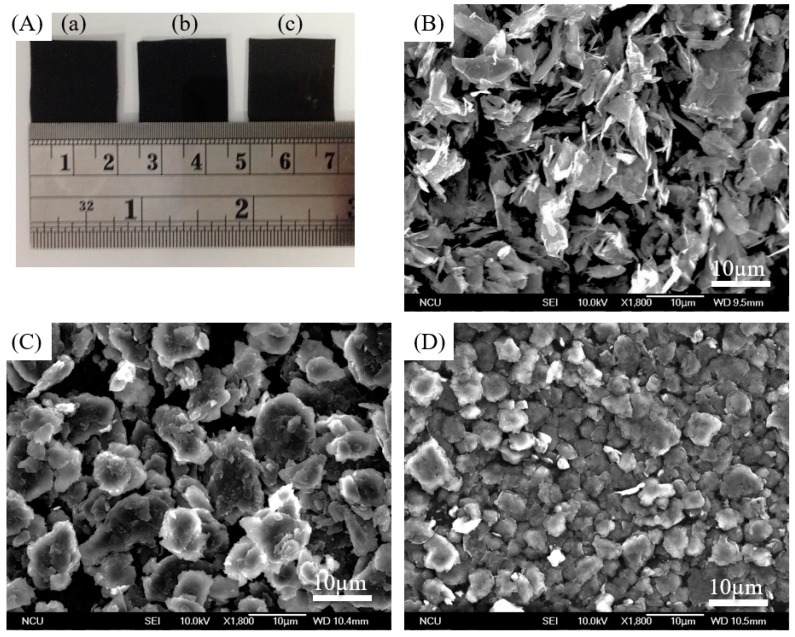
SEM images of EGN, recycled 2B and BMEGN. (**A**) Digital photos of (**A**-**a**) EGN, (**A**-**b**) recycled 2B, (**A**-**c**) BMEGN; (**B**–**D**) SEM images of (**B**) EGN, (**C**) ball-milled for 4 h recycled lead-graphite pencils (recycled 2B), and (**D**) ball-milled for 4 h exfoliated graphite nanoplatelets (BMEGNs).

Figure 4B–D shows the scanning electron microscopy (SEM) images of three types of graphite material such that the surface morphologies can be determined. Obviously, the mechanical disintegration of ball milling can effectively reduce the average particle size (which decreased gradually from 15.14 to 2.72 µm), suggesting that mechanically exposed edges were mechanically induced after ball-milling process and could potentially enhance the thermal conductivity of TIMs. Grain size was estimated by the open-source software ImageJ for the SEM images of all samples. The SEM result also shows that the recycled 2B with the lateral size of tens of micrometers can be reliably produced. Furthermore, a similar surface morphology was observed from both recycled 2B and BMEGN samples, suggesting the carbon-based materials may have similar thermally conductive characteristics. In summary, the effect of ball milling can produce materials with better dispersion as well as a less dense morphology with a reduced length in the micrometer range.

### 3.3. Raman Spectra

**Figure 5:**
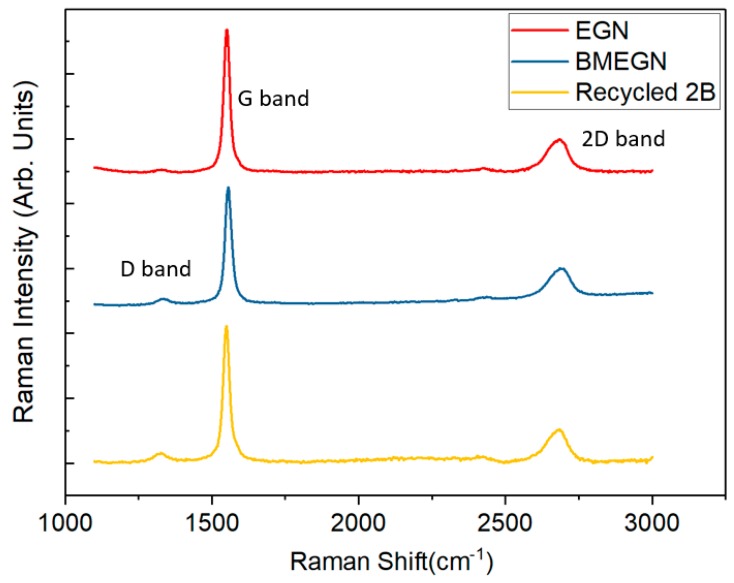
Comparison of Raman spectra (laser excitation 569 nm) for all three types of graphite sample.

Figure 5 shows the Raman spectra of EGN, recycled 2B and BMEGN samples, clearly indicating the distinguishable D-band, G-band and 2D-band (approximately at 1350 cm^−1^, 1580 cm^−1^ and 2700 cm^−1^, respectively). The spectral feature of graphene can be identified as the G-band (1580 cm^−1^) [39]. From the Raman spectra, no major change of spectral shape is observed for EGN, recycled 2B and BMEGN samples. In addition, the full widths at half maximum (FWHMs) of the G-band (1580 cm^−1^) are calculated as 27.2, 30.3 and 28.2 cm^−1^, respectively, for EGN, recycled 2B and BMEGN samples, indicating that the degree of graphitization is characteristically similar. Moreover, the previous work demonstrated that G and 2D Raman peaks could be affected by the number of graphene layers due to the evolution of the electronic structure and electron–phonon interactions [40].

### 3.4. Thermogravimetric Analysis (TGA)

**Figure 6:**
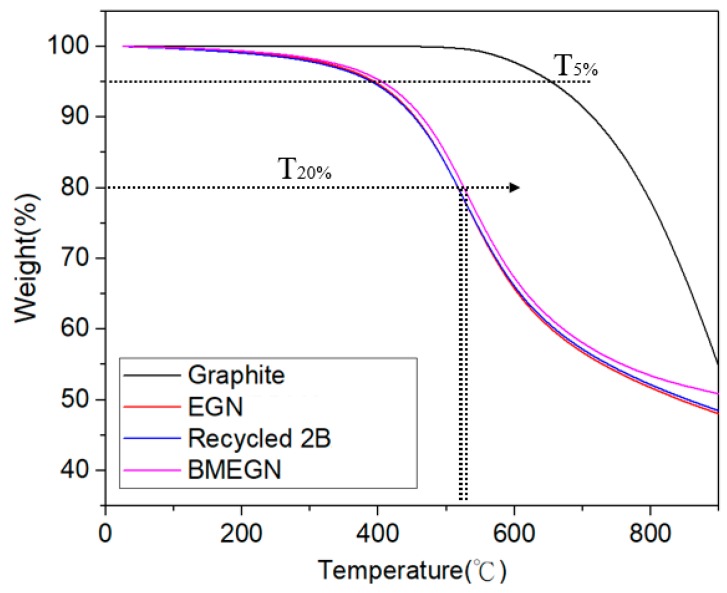
TGA curves of pure EGN, recycled 2B, BMEGN and graphite composites.

The thermal stability of the recycled 2B composite is shown in Figure 6, and the weight loss is less than 5% at 400 °C. All three types of graphite sample are thermally stable when heated up to 800 °C under inert atmosphere. Experimentally, the addition of BMEGN will certainly induce an increase of the thermal stability. Furthermore, the BMEGN composite displays better thermal stability than the EGN composite. For example, the corresponding temperatures *T*_20%_ (temperature at 20 wt % loss) are 527.6 °C, 516.4 °C and 515.9 °C for BMEGN, recycled 2B and EGN, respectively. This suggests that the BMEGN composite has the highest thermal stability of all tested samples. The recycled 2B composites pose a relatively higher heat capacity as compared to their EGN counterpart.

### 3.5. Thermal Conductivity (K) of BMEGN/PDMS Composite

**Figure 7:**
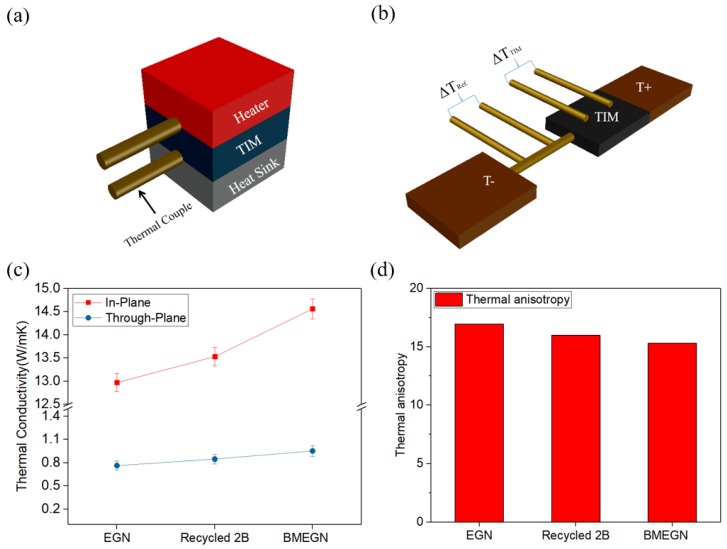
(**a**) Measurement setups of thermal conductivity in the through-plane (*K*_┴_) and (**b**) in-plane (*K*_//_); (**c**) measurement results of EGN, recycled 2B and BMEGN composites; (**d**) calculated anisotropy values (*K*_//_/*K*_┴_).

To quantitatively characterize the thermal conductivity of the fabricated TIMs, both through-plane (*K*_┴_) and in-plane (*K*_//_) directions are experimentally measured. Figure 7a shows a steady-state one-dimensional heat-conduction method (ASTM D5470-06) for the measurement of through-plane thermal conductivity (*K*_┴_) [37,38]. Figure 7b shows the in-plane thermal conductivity (*K*_//_) measurement setup [34]. Figure 7c shows the measurement results of the films. The *K*_//_ increases from 12.97 W/mK (EGN) to 13.53 W/mK (recycled 2B), as compared with the BMEGN samples with the highest in-plane thermal conductivity of 14.56 W/mK. The relatively lower thermal conductivity of recycled 2B samples (as compared to their commercially available BMEGN counterpart) is primarily attributed to impurities existing in the original 2B pencils: C (51.4%), Si (13.9%), O (20.3%), Fe (5.5%), Al (5.0%) and Ca (1.0%) [41]. On the other hand, the through-plane thermal conductivity (*K*_┴_) is comparatively small while the trend is the same, that is, the *K*_┴_ increases from 0.76 W/mK (EGN) to 0.84 W/mK (recycled 2B) and 0.95 W/mK (BMEGN) for ball-milled composites. Anisotropy in the thermal conductivities was experimentally observed (Figure 7d) as measured in the range of 15.31 to 16.95; the fundamental reason may be primarily attributed to the hierarchical structure consisting of PDMS matrix and aligned BMEGN [42]. Present measurements show comparatively small variation in anisotropy, irrespective of the different materials of EGN, recycled 2B and BMEGN composite.

### 3.6. Heat Dissipation Tests for High-Power IGBT

**Figure 8:**
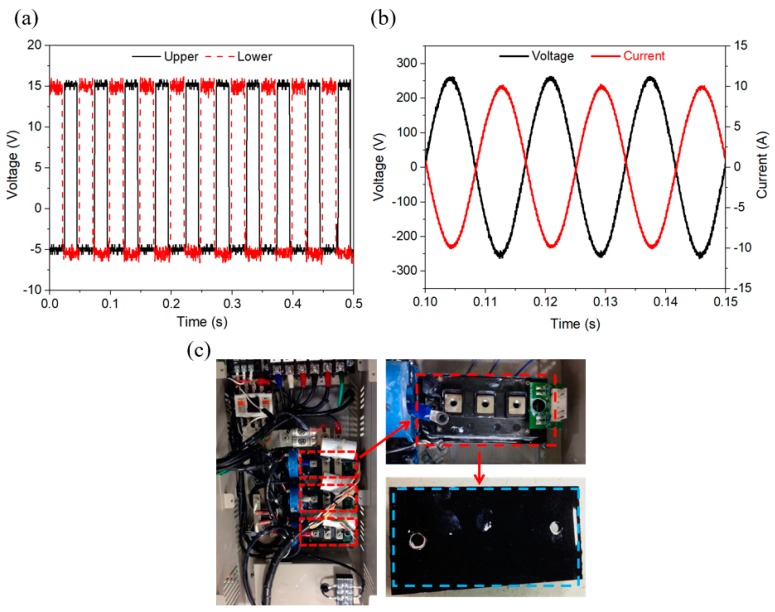
Inverter experiment operating at a frequency of 20 kHz pulse frequency. (**a**) *V*_GE_ voltage flow over an IGBT inverter for upper and lower arms; (**b**) measurement result of AC waveforms *V*_AC_, *I*_AC_; (**c**) TIMs (red dotted square) in measurements on the IGBT heat sources. Red rectangles show the 3 IGBTs in the normal operation position.

Figure 8a shows V_GE_ voltage transmits across an IGBT inverter module at the pulse frequency of 20 kHz. The current loading and the pulsing frequency are mimicking the operating conditions in renewable energy sources such as wind power stations. The designed IGBT specification of electrical output has a current loading of a maximum up to 100 A at 600 V. In the actual experiments of standalone operation, Figure 8b shows the experimental output voltage (*V*_AC_) and current (*I*_AC_), that is, the switching frequency/power at 60 Hz/2.0 kW, the output voltage is 270 V (*V*_p-p_ ~ 550 V) and output current is 9.8 A (*I*_p-p_ ~ 19.7 A). The output voltage and current perform a well-regulated sinusoidal waveform. As shown in Figure 8c, the developed BMEGN composites of TIMs (blue squares) are used to effectively conduct heat flow in the direction normal to the contact interface. Thermocouples were used for the measurements of transient temperature. Red rectangles show the three IGBTs where TIMs were sandwiched between the heat-generating IGBT chips and the heat sink.

**Figure 9:**
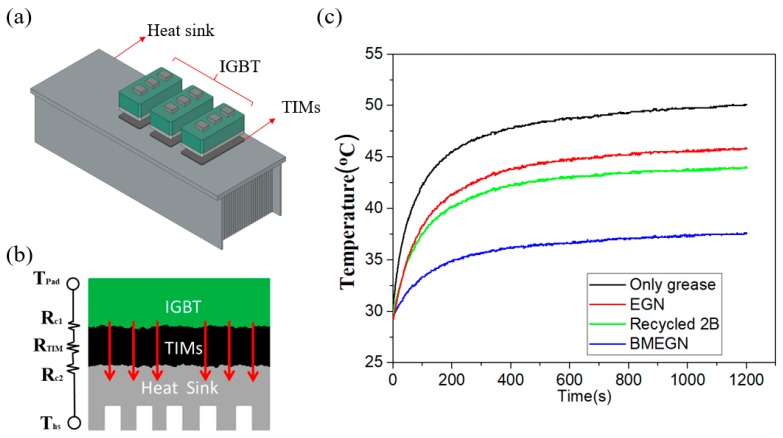
(**a**) Schematic setup of the heat dissipation test, showing three TIMs sandwiched between the IGBT chips and aluminum heat sink; (**b**) thermal resistance network; (**c**) temperature measurements of heat dissipation tests of EGN, recycled 2B and BMEGN composites.

Figure 9a presents a schematic of the heating test by sandwiching TIMs between the IGBT and aluminum heat sink. TIM samples (10 wt %) should have high thermal conductivity while being thick enough to be compliantly conformal to the unsmooth surface. In practice, the thin TIM layer between the contacting surfaces should be pressure-induced to underfill the gap, and the thermal conductivity-equivalent circuit model should include the combined effect of the thermal-interface resistances, labeled *R*_C1_ and *R*_C2_, respectively (Figure 9b, inset). Figure 9c shows the measurement results of temperature rise during the converter operation condition of 2.5 kW capacity. The experiment lasted 1200 s. Experimental results of temperature rise are specifically targeted for samples of BMEGN TIMs, recycled 2B TIMs, EGN TIMs and without TIMs (only grease). At 1200 s, the measured temperatures were recorded as 37.5 °C, 43.9 °C, 45.8 °C and 50.1 °C, respectively. The results indicate that the recycled 2B composite effectively reduced temperatures by approximately 6.2 °C as compared with the sample of grease alone. The recycled 2B composite is less effective than the BMEGN counterpart; however, it compares favorably with the EGN composite, demonstrating the feasibility of recycled processing of synthesis nanomaterials as an effective technique in reducing the fabrication cost in an environmentally benign way.

## 4. Conclusions

Graphite-based materials, such as lead-graphite pencils, have been widely used and discarded easily due to their low cost. The present work shows the commercial potential of thermally conductive recycled 2B composites that can be ball milled with homogenously dispersed fillers into the matrix and directly applied to TIMs. The thermal conductivities of composites with recycled 2B filler were characterized by SEM and TGA. The mechanical (bending test) and thermal performance of three types of carbon filler (EGN, recycled 2B and BMEGN) dispersed in epoxy resin were experimentally investigated. The recycled 2B is a renewable low-cost source, and ball milling can transform the initial bulk morphology into a spherical form. Therefore, the thermal conductivity of recycled 2B can be enhanced via uniformly dispersed particles (increasing the specific surface area). Fundamentally, ball milling can be effective in enhancing the mechanical mixing processes between a polymer matrix and embedded recycled 2B, such that filler agglomerates can be fragmented segments with a homogeneous dispersion inside the matrix. The measured in-plane thermal conductivity (*K*_//_) increases from 12.97 W/mK (EGN) to 13.53 W/mK (recycled 2B, enhanced by 4%) after four hours of ball milling. In summary, ball milling offers the potential of preparing TIM composites at low cost and with an easily scalable method. For the IGBT tests, the results indicate that the composite of recycled 2B is capable of effectively reducing temperatures by approximately 6.2 °C as favorably compared with thermal grease. This work provides new insights into the relationship between sample preparation methods and the formation of highly efficient conductive paths of polymer composites.
